# The Essential Co-Option of Uracil-DNA Glycosylases by Herpesviruses Invites Novel Antiviral Design

**DOI:** 10.3390/microorganisms8030461

**Published:** 2020-03-24

**Authors:** Renos Savva

**Affiliations:** Institute of Structural and Molecular Biology, Department of Biological Sciences, Birkbeck, University of London, Malet Street, London WC1E 7HX, UK; r.savva@mail.cryst.bbk.ac.uk; Tel.: +44-0-20-7631-6805

**Keywords:** herpes, herpesvirus, uracil-DNA glycosylase, Ung, antiviral, drug discovery, novel chemical entity

## Abstract

Vast evolutionary distances separate the known herpesviruses, adapted to colonise specialised cells in predominantly vertebrate hosts. Nevertheless, the distinct herpesvirus families share recognisably related genomic attributes. The taxonomic Family *Herpesviridae* includes many important human and animal pathogens. Successful antiviral drugs targeting *Herpesviridae* are available, but the need for reduced toxicity and improved efficacy in critical healthcare interventions invites novel solutions: immunocompromised patients presenting particular challenges. A conserved enzyme required for viral fitness is Ung, a uracil-DNA glycosylase, which is encoded ubiquitously in *Herpesviridae* genomes and also host cells. Research investigating Ung in *Herpesviridae* dynamics has uncovered an unexpected combination of viral co-option of host Ung, along with remarkable Subfamily-specific exaptation of the virus-encoded Ung. These enzymes apparently play essential roles, both in the maintenance of viral latency and during initiation of lytic replication. The ubiquitously conserved Ung active site has previously been explored as a therapeutic target. However, exquisite selectivity and better drug-like characteristics might instead be obtained via targeting structural variations within another motif of catalytic importance in Ung. The motif structure is unique within each Subfamily and essential for viral survival. This unique signature in highly conserved Ung constitutes an attractive exploratory target for the development of novel beneficial therapeutics.

## 1. Introduction

In this article, the emphasis will be on focusing evidence to encourage structure-based drug design activity on the conserved uracil-DNA glycosylases encoded by diverse herpesviruses. The specific differences between the uracil-DNA glycosylase enzymes encoded by herpesviruses subfamilies are important in this regard. It is also worth considering these differences as compared to the host-encoded version of the enzyme. Drug discovery targeted at the much more highly conserved active site is less interesting from that fact alone, but also since drug-like properties are the key.

The context is that although there are many successful therapeutic agents indicated for herpesvirus treatment, their use in more challenging healthcare settings is found unsatisfactory, particularly in regard to immunocompromised patients (i.e., autoimmune-suppressing medication, transplant patients, and those with underlying health conditions, such as those living with HIV). The major problems are toxicity and, to some extent, resistance phenomena, which in healthier patients, would be self-limiting. It is acknowledged that many advanced therapies and vaccination approaches are at the forefront of antiherpetic therapy research, but the role of small molecules in certain healthcare contexts could easily prove more cost-effective at scale.

Previous efforts at drug discovery, specifically targeting the HSV-1 UL2 gene product, are presented as a contrasting approach to the de novo tactics suggested herein, i.e., from the perspective of considering the properties most attractive in a lead compound. To support the notion of a new target focus within the catalytically important structural features of these otherwise very well conserved enzyme domains, the biological strategies of viruses, in general, to avoid uracil-DNA glycosylases are considered. In contrast, the alternative way in which herpesviruses have dealt with innate immune factors in order to retain a working relationship with uracil-DNA glycosylases is also presented. In herpesviruses, the adaptations to the viral copy of the enzyme are subfamily-specific, and suggest potentially more drug-like exquisite specificity than via targeting of the very well conserved enzyme active site. Targeting this area, it is suggested, will be specific to a herpesvirus subfamily and quite separate also from the host enzyme in this region of the molecular structure.

## 2. Herpesviruses

Herpesviruses of the taxonomic Order *Herpesvirales*, are ancient virions with extensive genetic repertoires in genomes ranging in length from 0.1 to 0.3Mb (data according to NCBI Viral Genomes at the time of writing; see, e.g., NC_001987 and NC_009127). Herpesviruses under discussion here belong to the taxonomic Family *Herpesviridae*. *Herpesviridae* are commonly classified into three major taxonomic Subfamilies: *Alphaherpesvirinae* (e.g., in humans: HSV-1, HSV-2, VZV), *Betaherpesvirinae* (e.g., in humans: hCMV, and *Roseoloviruses* 6A, 6B, and 7), and *Gammaherpesvirinae* (e.g., in humans: EBV, and KSHV)† († Colloquially referred to in this work as, respectively: alphaherpesviruses, or via the symbol α; betaherpesviruses, or via the symbol β; gammaherpesviruses or via the symbol γ.) (Table 1) [1].

In terms of tropism, the range of specialised cell types that herpesviruses have adapted to enter and propagate within is broad: neural, muscular, epithelial, and immune system cells of various types. Such tropism is a function of the enormous length of time that these viruses have interacted with and adapted to their hosts and is no doubt an underlying reason for their endemic success. Regardless of the subfamily, but dependent upon the type of cell involved, herpesviruses will exhibit two discrete cellular phases: the lytic phase, and the latent phase. In their endemic state, herpesvirus genomes are observed to be predominantly latent: usually residing as epigenomic circular DNA in vertebrate cell nuclei [2].

The lytic phase comprises viral entry and expansion through replication: thus permitting spread of virions to neighbouring cells. This pattern of infection resulted in the archaic naming of these viruses: The Greek word “herpes”, refers to “slow-moving creeping” and is cognate of the Latin “serpere” the sense of a “snake-like creeping” [3], both of which describe the appearance of the propagative lytic spread of shingles or common cold sores. Such appearance is, therefore, descriptive of *Alphaherpesvirinae* lytic phase replication and cellular egress visible on human external epithelia.

In latency, the viral genome undergoes stable nuclear sojourn in the form of episomal circular DNA; except, however, in the betaherpesvirus *Roseoloviruses* HHV-6A and HHV-6B, which in ~1% of the human population is integrated into host chromosomes by homologous recombination at predominantly the sub-telomeric regions, and are also thus capable of being passed on in the germline [4,5]. Viral episomes can reactivate via sensing of organismal environmental triggers, to enter the lytic phase and thereby intercellular virion propagation (i.e., pathogenesis), which can occur periodically during the lifetime of a host. These characteristics of virus-host interactions ensure that herpesviruses remain endemic among host populations. Estimates of infection probability with *Herpesviridae*, during human lifespans across several decades, approach saturation in endemic populations for the best-studied human pathogenic types [1,2].

## 3. Herpesviridae and Human Health Considerations

Due to their propensity to reactivate from latency, *Herpesviridae* in humans are frequently pathogenic in a periodically acute sense. Certain pathogenic outcomes are disabling episodically, resulting in either essentially complete functional recovery, or else permanent damage to cells or tissue structures. There is a risk of fatality due to the primary effects of herpesvirus infection such as oncogenesis, encephalitis, oedema or haemorrhage. There can also be secondary effects: For example, morbid systemic complications, or bacterial superinfection; in the worst cases these secondary issues can also result in fatality [1,2,4,5,6,7].

In modern healthcare settings, depending to some extent upon local factors, herpesviruses present important post-surgical and tissue-transplant complications that are imperfectly controlled with currently available therapies. The nature of herpesvirus pathogenicity problems is that although the viruses are endemic at high levels in many populations, their life-threatening etiologic presentation is sporadic; therefore, serious cases would normally manifest in numbers more on a par with orphan diseases [2]. 

Exacerbation of the pathogenic effects of herpesviruses can be due to underlying health conditions, or else iatrogenic in particular healthcare settings requiring immunosuppression. One mode of acquisition of a new infection is via transfer of infected donor material in situations such as transfusion and transplant [2,5]. Screening and treatment of donor material can significantly lower the risk of new infection via this route. However, extant infections can be caused to flare up or to become chronically problematic via immunosuppression: For example, to suppress autoimmune indications, or to permit transplant of matched donor tissue.

In most situations, treatment with current antivirals indicated for that specific use will result in the successful management of herpesvirus infections [2,5,6]. General success has thus been sufficient to curtail urgent interest in the financially risky extensive development of novel antivirals targeting orthogonal weak points in the replicative cycles of these viruses. The risk of going after new targets is exemplified by the recent disappointing phase III clinical trial results with marivabir, in spite of encouraging phase II data [8].

The range of successful antiherpetics historically approved for use in therapy, nevertheless, targets different replicative stages in the herpesviruses. The major deployed drugs include acyclovir, famciclovir, ganciclovir, cidofovir and letermovir [2]. Notwithstanding the success of these drugs, there are toxicity effects and resistance phenomena in the immunocompromised/ immunosuppressed population [1,2,4,5,6,7]. Therefore, alternative therapeutic agents that could also lower the viral load or reduce the viability of virus progeny, but that act in novel modes with less toxicity compared to those currently available, would improve control of these important viral pathogens. This may, analogously to drugs developed to treat orphan diseases, be worth the financial risk: Particularly in special healthcare settings, due to recognised problems with current treatment outcomes.

## 4. Uracil in DNA and its Relevance to Viruses

**Figure 1:**
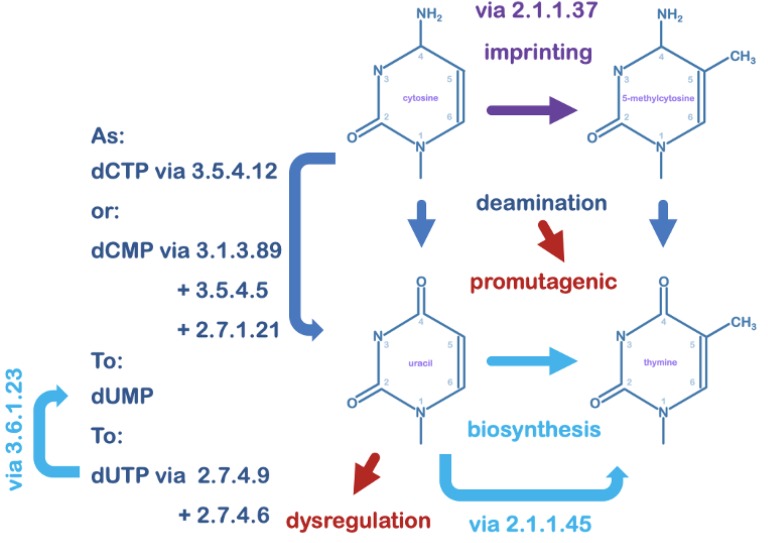
DNA pyrimidines and their interconversion. The pyrimidine uracil as a deoxynucleotide is a precursor in thymidine biosynthesis. Deoxyuridine can accrue in DNA by misincorporation under conditions of nucleotide pool perturbation and imbalance such as during viral replication; its unique structure can create dysfunction in gene regulation. The DNA base cytosine is also converted to 5-methylcytosine in epigenetic imprinting. Both cytosine and 5-methylcytosine can convert in situ to uracil and to thymine, respectively, via spontaneous loss of the 4-amino group under ambient cellular conditions. This conversion in the context of the original base pair is considered promutagenic and must be repaired to retain sequence fidelity upon replication. Enzymes listed by KEGG Orthology are: 2.1.1.37 (cytosine-5)-methyltransferase; 2.1.1.45 thymidylate synthase; 2.7.1.21 thymidine kinase; 2.7.4.6 nucleoside diphosphate kinase; 2.7.4.9 thymidylate kinase; 3.1.3.89 HD-domain 5′-nucleotidase; 3.5.4.5 cytidine deaminase; 3.5.4.12 dCMP deaminase.

It has been considered that the primary occurrence of uracil in DNA in a normal cell is via spontaneous deamination of cytosine bases under ambient cellular conditions; an event estimated to occur around 600 times per day per human cell [9]. Environmental mutagens such as bisulphite can appreciably accelerate the number of deamination events [10]. Failure to act upon uracil thus occurring in DNA, would result in the transition of a C:G base-pair to an A:T base-pair upon replication. Cytosine deaminates at an accelerated rate in single stranded DNA [11,12], thus too 5-methyl cytosine converting to thymine, which thus appears as a mismatched base in the context of the canonical cytosine base pair with guanine. The spontaneous T:G mismatch is as promutagenic as spontaneously arising DNA uracil (Figure 1; Table 2) [13,14].

Viruses can, due to their population sizes during replication, sample fitness via mutation [15]. Nevertheless, coding sequences and regulatory motifs are at risk if excessive levels of mutation should accrue [16]. There is, however, mounting evidence from recent uracil-sequencing technologies that DNA uracil may be a managed signal for tissue-specific gene regulation [17]. In that case, it is possible for relevant cellular programs utilising uracil to be usurped by viruses to their advantage or/and to the detriment of the cell. Certainly, herpesviruses orchestrate large-scale degradation of host nucleic acids and rapid biosynthesis of progeny, managed with their own encoded nucleotide biosynthesis and DNA uracil-excision related proteins.

Uracil can also be misincorporated by DNA polymerases, which do not appreciably distinguish between deoxyuridine or thymidine nucleotides (exceptions are DNA polymerases of archaea, and of some bacteriophages). A deoxyuridine misincorporation event is insured against in normal cells due to the nucleotide biosynthesis pathways biasing heavily against deoxyuridine triphosphate through the action of dUTPase enzymes, and the diversion of dUMP to thymidine biosynthesis [18]. Relevant to the focus of this article, it should be noted that nucleotide pool imbalances, in general, contribute to increased error rates by DNA polymerases and that viral replication is likely to perturb this [19]; also, viral DNA polymerases tend to exhibit lower replicative fidelity than cellular counterpart enzymes [20]. A dUTPase is, in fact, encoded by *Herpesviridae*, although in betaherpesviruses it is apparently not functional, but potentially, the phenotype is rescued by another viral protein [21]. Uracil in a regulatory sequence of a gene could potentially deleteriously modulate the affinity of gene regulatory factors [22,23,24] because unlike thymine it lacks a 5-methyl group; thus, some insurance against uracil occurring in DNA would benefit virus progeny fitness.

The leftmost column indicates the pyrimidine base in the context of its canonical base pair within a DNA duplex. The second column indicates natural cellular states that afford some insurance against uracil substituting for the canonical pyrimidine: DNA in a canonical duplex, or when bound to proteins that do not unwind it into single strands, is protected in that rates of cytosine deamination are reduced. In addition, dUTPase keeps the levels of dUTP low enough that misincorporation by DNA polymerase is negligible under normal cellular functioning conditions; cellular replicative polymerases are high-fidelity enzymes, and this is yet another protective measure that normally keeps uracil from being introduced as a partner base for adenine. The third column indicates the state of the pyrimidine after substitution in the context of its base partnering either as a pair or mismatch: The U:A base-pair is most likely to interfere with gene regulation primarily due to uracil lacking a methyl compared to thymine. The mismatches of uracil or thymine with guanine could lead to regulatory dysfunction, or permanent mutations unless repaired, as indicated in the penultimate column. In the final column on the right, UDG means primarily Ung, but it could also be some other uracil-DNA glycosylase superfamily enzyme type under certain circumstances. TDG is a [UDG-superfamily] thymine-DNA glycosylase.

## 5. Uracil-DNA Glycosylase in the Maintenance of DNA Integrity

**Figure 2:**
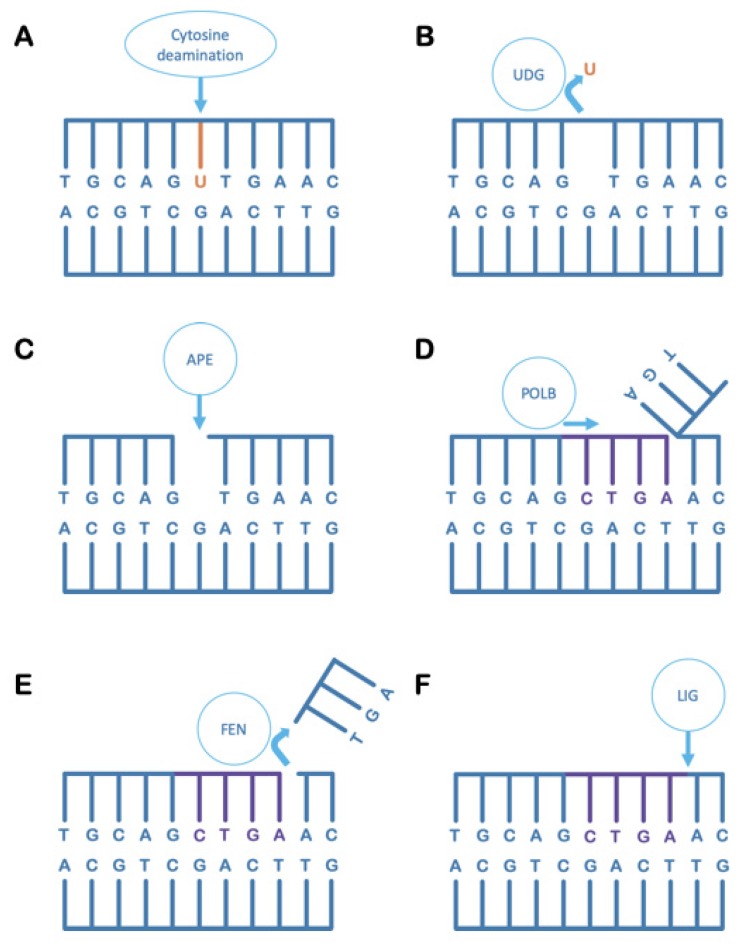
The base excision repair pathway. (**A**) Uracil spontaneously occurs in DNA due to the deamination of cytosine bases. If left uncorrected, the uracil-containing strand will give rise to a permanently mutated daughter strand upon replication. (**B**) A uracil-DNA glycosylase (UDG) cleaves the uracil base from the DNA backbone, leaving an abasic site in the DNA. (**C**) An AP-endonuclease (APE) cleaves the phosphodiester backbone, 5′ of an abasic site [AP refers to apyrimidinic, or lacking a pyrimidine; it can also refer to apurinic in other contexts]. (**D**) A type-B DNA polymerase (POLB), typically involved in short-patch DNA repair, resynthesises several bases complementary to the undamaged strand while displacing the cleaved strand beginning at the nick generated by AP-endonuclease. (**E**) A Flap-Endonuclease (FEN) cleaves off the displaced DNA strand after the B-type DNA polymerase has dissociated, leaving behind a nick in the DNA duplex. (**F**) A DNA ligase (LIG) seals the nick to return DNA to its canonical pre-damaged state.

Uracil-DNA glycosylase (UDG) is a name that describes a superfamily of DNA base excision repair-initiating enzymes that share a common protein structural fold and related active site architecture [25]. UDG enzymes primarily recognise uracil in DNA, the occurrence of which is potentially promutagenic, and act to specifically remove uracil bases by cleavage of the N-glycosyl bond to the deoxyribose; this produces an abasic site in the DNA. A sequential process of action by other enzymes is thus triggered, in the so-called base excision repair (BER) pathway, which returns the DNA chemistry to its canonical state to maintain DNA sequence fidelity (Figure 2).

## 6. Uracil-DNA Glycosylase Can Also Contribute to Irreversible DNA Damage

**Figure 3:**
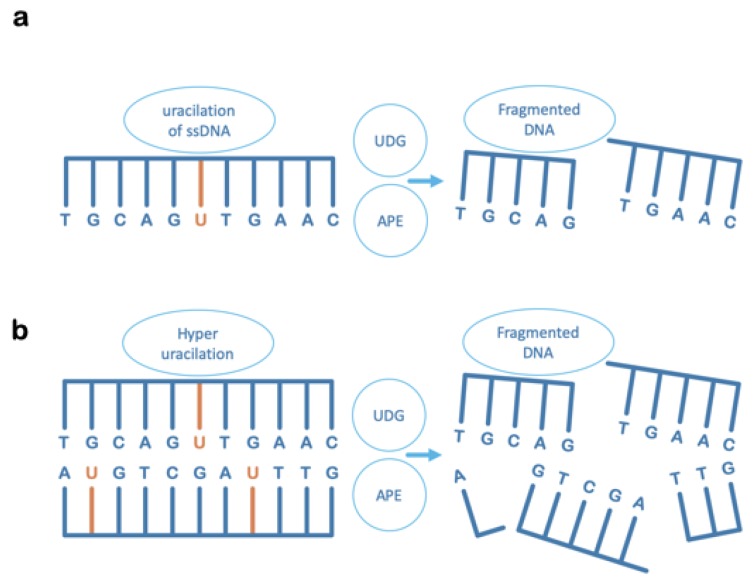
UDG can contextually disrupt DNA integrity. The destructive effect on DNA chain integrity after the action of uracil-DNA glycosylase (UDG) and AP-endonuclease (APE) during initiation of BER, on uracil-containing DNA in situations where: (**a**) Uracil occurs in a single-stranded DNA molecule, in which case there could be stalling or disruption of DNA replication, or of ssDNA-transfer processes such as in bacterial conjugation or in virus replication or packaging. (**b**) There are unusually high levels of uracil in duplex DNA, in which case backbone breaks will be enzymatically created on both strands of dsDNA at close proximity. At short oligonucleotide lengths, the hydrogen bonding forces between complementary base pairs are insufficient to resist the thermal disintegration of the duplex, and the consequence is chain fragmentation that is incompatible with high-fidelity DNA repair.

The occurrence of multiple, proximal uracil residues spaced at oligonucleotide lengths from each other on opposing strands, would cause the initial steps of the BER pathway to create double-strand breaks with the loss of intervening oligo-length sequences (Figure 3) [26]. Although high concentrations of uracil bases in DNA would not occur naturally under ambient conditions, fascinatingly, this is something that can be enzymatically induced by cellular factors. The situations where enzymatic conversion of DNA cytosine to uracil is known to occur, are: (1) Following sensing of virus DNA in the cytoplasm [27,28]; and (2) under programmed conditions in the humoral immune response [29,30]. In addition, relevant as regards unusually high concentrations of DNA uracil: Viruses such as HIV-1 appear to involve a uracil-DNA stage prior to retroviral integration [31,32,33]; and, certain bacteriophages comprise thymine-free uracil-DNA genomes [34,35,36,37]. In all cases, cellular Ung in concert with AP-endonuclease would act as a potent restriction enzyme upon invading uracil-rich pathogen DNA [38].

## 7. The Ung-Type Uracil-DNA Glycosylase Is Central to the Host Pathogen Response

A UDG was the first described enzymatic activity involved in a process of overt DNA damage repair [39]. The UDG described in those initial studies is known as Ung, and is ubiquitous in the major kingdoms of life (N.B. excluding archaea, which instead employ other branches of UDG superfamily enzymes). In fact, the ubiquity of Ung extends to its appearance in the genomes of some viruses: For example, Ung is encoded by poxviruses [40,41], and herpesviruses [41,42,43,44,45,46,47,48,49,50,51,52].

**Figure 4:**
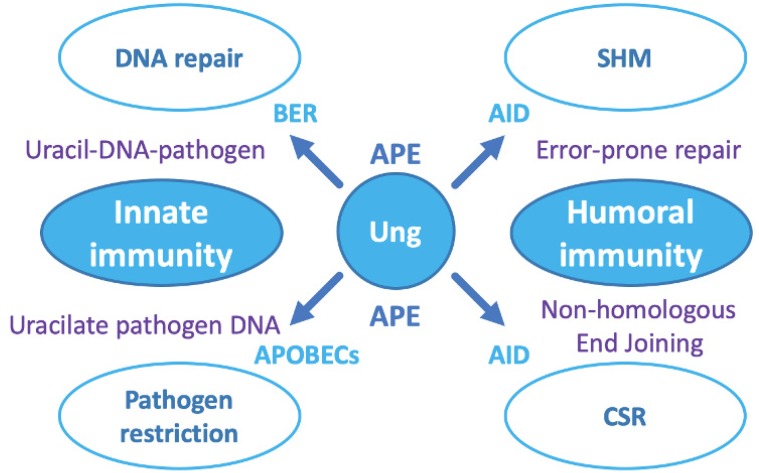
The roles of Ung in cellular programs. On the left side of the schematic: Innate cellular immunity. Ung acts upon spontaneously arising or enzymatically generated uracil residues. For random chemical events, Ung initiates DNA repair by base excision repair (BER); in the pathogen response, UDG follows APOBEC3 enzymes that deaminate cytosine in pathogen DNA to cause hyperuracilation. On the right side of the schematic: Development of humoral immunity. Ung acts downstream of the cytidine deaminase AID, which generates uracil residues in (i) Immunoglobulin variable chain regions to initiate somatic hyper mutation (SHM), generating variable chain diversity; and (ii) immunoglobulin class switch regions to initiate class switch recombination (CSR), permitting successful antibody variable chains to be deployed via alternative antibody scaffolds, e.g., IgM to IgG. The role of Ung and APE are the same as in BER, but the different pathways (error-prone repair, or non-homologous end joining) deploy alternative suites of proteins downstream to create the intended outcome.

Cellular utilisation of Ung activity is not limited to its role in DNA repair (Figure 4), thus Ung is observed to play important roles in other key cellular programs, such as innate cellular immunity as a frontline defence against viral pathogens. The importance of this role for Ung is underlined by the fact that diverse virus lineages, whether targeting prokaryotes or eukaryotes, actively silence Ung at the mRNA or/and at the protein level [41,53,54,55,56,57,58,59,60]. It would appear that viruses, in general, are sensitive to uracil in DNA for varying reasons. Some retroviruses, such as HIV-1, apparently strategically manage uracil-DNA to efficiently integrate into host genomic DNA and must, therefore, silence as well as co-opt Ung [31,32]. Other viruses, as observed in unrelated lineages of bacteriophages, generate single-stranded DNA intermediates during replication or assembly [60,61,62,63]. Naked single-stranded DNA is at much higher risk of cytosine deamination than either protein-coated single strands [64], or duplex DNA [11]. Furthermore, BER acting at a randomly occurring uracil residue in single-stranded DNA would create deleterious strand breakage upon the action of AP-endonuclease in the wake of Ung activity (Figure 3a).

Ung acts as part of both the innate and humoral immune response pathways downstream of AID and APOBEC (Apolipoprotein B mRNA Editing Catalytic polypeptide-like family) enzymes, which enzymatically deaminate cytosine in DNA [65,66]. In the innate response, this is triggered by pathogen DNA detection in the cytoplasm. Interestingly, herpesviruses have been reported to antagonise APOBEC3 and thus will not succumb to restriction by Ung [67,68]. This property would enable herpesviruses to utilise Ung for its more beneficial properties in DNA repair, but does not in itself reveal why they encode their own, adapted copy. In other types of virus, appreciable accumulation of DNA uracil in the wake of APOBEC activity will result in base-excision by Ung in close proximity on both strands of the affected DNA and thus pathogen DNA fragmentation due to BER-induced double-strand breaks (Figure 3b; Figure 4). In bacteria, Ung is present residually and is therefore a potent restriction enzyme against viral DNA that accumulates uracil as it is rapidly expanding (i.e., due to relatively lower fidelity of viral DNA polymerases and the effects of nucleotide pool bias) especially if utilising single-stranded intermediates [59,60,61,62,63]; as mentioned earlier, viruses employing uracil as a substitute for thymine are a prime target for Ung [38].

Ung plays a similar role in the maturation of the humoral immune system at the genetic level. This is in the context of two essential processes: (a) Somatic Hypermutation [29], in which antibody variable domains are created, and (b) class switch recombination [30], in which antibody heavy chain types can be swapped for successfully selected antibody variable domains. Ung acts subsequent to activation-induced deaminase (AID), which first enzymatically converts cytosine to uracil under strictly controlled conditions: Error-prone repair follows in SHM, while non-homologous end joining follows in CSR, to complete these molecular processes (Figure 4).

## 8. Herpesviruses and Ung, a Surprising Relationship and Remarkable Exaptation

**Figure 5:**
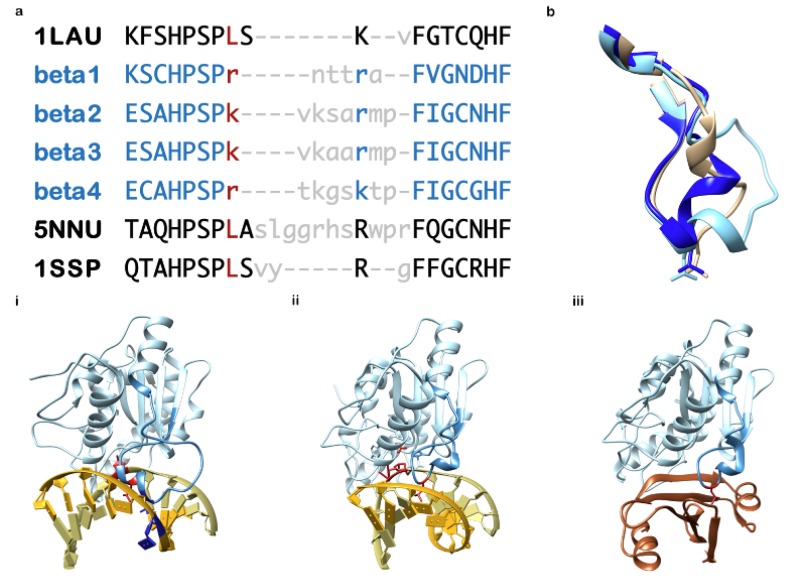
Sequence and structure alignment of the herpesvirus adapted motif central to Ung catalysis. (**a**) Multiple sequence alignment of the Ung minor groove DNA intercalation loop motif, generated via structural superposition of protein chains: UNG2 (Human), UL2 (HHV-1), and ORF46 / HHV8GK18_gp50 (HHV-8), using the program Chimera: PDB accession codes are used (UNG2 = 1SSP; UL2 = 1LAU; ORF46 = 5NNU, chain A). Ung sequences for which there is currently no deposited molecular structure (i.e., β-herpesviruses) are indicated in blue font (beta 1 = HHV-5; beta 2 = HHV-6A; beta 3 = HHV-6B; beta 4 = HHV-7). Residue positions lacking structural equivalence are shown in lower case with gross differences in grey font. The Ung catalytic leucine residue is in dark red font as are (in lower case) positionally equivalent betaherpesvirus residues. (**b**) Cartoon structure excerpts of the aligned region only, overlaid (from 1LAU, 1SSP and 5NNU). (**c**) The sequence aligned is displayed as a darker shaded region of the entire Ung molecule in its biological contexts: (**i**) 5NNU: ORF46 (HHV-8) Ung [chain A] in complex with dsDNA [chains S and T] (**ii**) 1SSP: UNG2 (Human) [chain E] in complex with dsDNA [chains A and B], (**iii**) 1UDH: UNG2 [chain E] in complex with Ugi [chain I], a protein mimic of DNA that specifically targets Ung. Highlighted in dark red is the Ung catalytic leucine (i),(ii),(iii) and catalysed DNA residue (i),(ii); highlighted in dark blue (i) and unique to gammaherpesvirus Ung is a key residue of an exaptation that precisely deforms DNA (also highlighted dark blue) as a possible signal essential to viral DNA replication assembly in gammaherpesviruses: This novel exaptation, shown for 5NNU (HHV-8 Ung), is conserved in HHV-4 (deposited as pdb structure 2J8X – not shown).

An operational Ung gene is, as described, essential to cellular survival. Typically Ung catalytic domains are highly conserved in protein sequence and structure, and particularly so in the active site. It is probably not surprising to consider that due to the role of Ung in innate cellular immunity against viral pathogens, a diversity of invading virus types sequester Ung predominantly to silence it. It is, therefore, somewhat enigmatic to consider that all known herpesviruses encode an Ung, and that, furthermore, at least the gammaherpesviruses also additionally utilise the host cell encoded Ung [69]. If that is not surprising enough, γ-herpesvirus Ung includes the exaptation of a key motif essential for Ung catalysis to underpin viral replication competence (Figure 5) [49,50,51,52]. The aforementioned Ung catalytic motif is, in fact, the same one that, in the host Ung and its canonical relatives in bacteria, is targeted by viruses that silence the cellular Ung protein as part of their replication strategy (Figure 5) [70,71,72].

Ung substrate search and engagement for catalysis involves initial docking with DNA by electrostatic alignment and then a subtle distortion of the duplex upon complex formation. The distortion induced in DNA by the unusual shape of the Ung DNA binding surface both elicits and prolongs a property of dynamic cellular DNA known as breathing [73]. DNA breathing is a natural phenomenon of base pairs fleetingly breaking in response to DNA shape modulation: In the aqueous cellular environment, DNA is subject to continuous molecular impact forces that are translated into motion. In addition, protein complex formation at any point will induce subtle or gross structural manipulation of the DNA duplex, this is translated along the DNA axis to dissipate the strain forces. All of these events are also contextual upon the local DNA sequence composition. The result is that base pairs in duplex DNA not only tilt or twist to remain hydrogen bonded, but those bonding forces are also often overcome, which induces natural DNA breathing; under normal cellular conditions thermal forces are considered constant.

Upon complex formation with Ung, the DNA duplex is pinched to produce mild deformation and will thus elicit breathing events in weakly paired bases, such as A:T/A:U pairs or G:U/G:T mismatches. Prolongation of this Ung-induced DNA breathing occurs when a mobile component of the Ung structure, known as the minor groove DNA intercalation loop, automatically swings in upon complex formation, displacing the pyrimidine nucleotide into the active site vicinity. A breathing base pair with adenine or a mismatch with guanine will be overpowered by the concerted motion of this loop. The apical residue on the loop, now interior to the DNA helix, will form a pseudo-base pair with the purine. The loop apical residue is most usually a leucine side chain, may rarely be phenylalanine, and is potentially a lysine or arginine in a betaherpesvirus Ung).

Entry to the Ung active site for the flipped-out pyrimidine is carefully screened. A peripheral external cavity would exquisitely capture the 5-methyl moiety of a thymine base to sterically avert the catalysis of canonical DNA. Consequently, Ung is unable to initiate repair of a G:T mismatch [this is the role of other DNA repair enzymes]. A cytosine base is chemically incompatible with the passage into the active site cavity. Exquisitely in terms of substrate selectivity, uracil is able to enter the active site, where hydrolysis of the N-glycosyl bond is immediate. Although extended in duration when compared to natural DNA breathing events, the residence time of the pseudo base pair is finite, and the Ung complex with DNA will rapidly dissociate, thus any free uracil base could leave the active site after catalysis. Importantly, an abasic site in duplex DNA is also promutagenic, thus the ability of Ung to easily bind back there will also attract downstream proteins [43,74,75].

Relevant to gauging the importance of Ung to virus replication strategies, the concerted motion of the aforementioned Ung loop and the aliphatic property of the apical residue, are targeted by diverse anti-restriction proteins encoded by unrelated viruses. The virus-encoded inhibitor proteins target Ung by amino acid mimicry of DNA contacts, arrayed in the unusual pinched shape normally induced in DNA by Ung. Intriguingly this conserved mechanism has evolved convergently from three independent, unrelated protein architectures: Ugi [and its structural homologue SAUGI, a horizontally transferred gene found in SCCmec mobile genetic elements of Staphylococcaceae] from myoviruses, p56 from salasviruses and Vpr from primate lentiviruses. Charge-based alignment and contact from the inhibitor protein elicits concerted Ung loop motion, resulting in an effectively irreversible sterically-blockaded sequestration of Ung via hydrophobic trapping of the apical aliphatic side chain of the Ung loop by the virus inhibitor protein (Figure 5) [41,70,71,72,76].

The minor groove DNA binding loop sequence itself, and its interaction with the rigid part of the Ung catalytic domain, is quite sequence variable in both length and residue type. This is in contrast with the very well conserved active site pocket, which has been the focus to date, of novel chemical entities for inhibition of Ung.

The loop sequence in the gammaherpesvirus Ung is moreover an exaptation related to virus lytic replication initiation. It is disordered but takes up a conserved structure (i.e., a structure that is conserved in both Ung of HHV-4 and HHV-8) upon binding of DNA or of a viral protein inhibitor. Unusual sequence deviations of a probably different nature are seen in the same loop in β-herpesviruses. Although not investigated in as much detail at the present time, structural molecular insights could also provide the potential for similar approaches to selective inhibition. This key motif in Ung, is, therefore, a promising target for the design and development of novel antiviral compounds [41,76], especially given the structural differences relative to the host cell enzyme (Figure 5).

## 9. Uracil in DNA and Its Potential Significance for Herpesviruses in Latency

In quiescent cells harbouring latent herpesvirus genomes, significant uracil in viral DNA could accumulate over long periods. Such DNA would be at risk of irreparable breakage should it encounter Ung activity upon reactivation, which is ironic given this would most likely be the virus encoded Ung. Therefore, although host Ung would be present continuously in active cell types to routinely prevent accumulation of uracil in viral DNA, the virus-encoded Ung would need to be maintained in cell types that are quiescent [24,42,77]. Interestingly, it would appear that the different Subfamilies of *Herpesviridae* have been able to adapt their use of the host or viral Ung, to suit the cell type in which latency is maintained.

## 10. Tropism of Herpesviruses as a Factor in Consideration of Targeting Ung

Taking into consideration the types of cells that harbour or spread herpesviruses may be radically different in their states of activity or quiescence, the potential role of Ung in these processes may also need to vary. In *alphaherpesvirinae*, lytic replication occurs in active epithelial cells, but latency is in the quiescent neuronal cells of peripheral sensory ganglia. In the *betaherpesvirinae*, cell tropism is broad with organs, glands, neuronal, epithelial and immune cells providing detectable virus presence during pathogenesis. It is surmised that the immune cells, such as monocytes, macrophages, and CD4+ T lymphocytes are the sites of latency, due to pathological effects of replication being seen in the other mentioned tissue types. Similarly, in *gammaherpesvirinae*, tropism is broad, majorly involving epithelia and immune system cells, but also smooth muscle. Latency and reactivation in *gammaherpesvirinae*, appears to be possible in a variety of cell types [1]. Of interest, viral Ung is essential in murine gammaherpesvirus 68 depending upon the level of cellular Ung activity. In cells such as lung, where host Ung activity is relatively low, the supplemental activity of the viral enzyme is essential for replicative fitness [77].

## 11. The Reported Roles of Ung in Herpesvirus Fitness

Ung, in alphaherpesviruses, is a product of the UL2 open reading frame [78]. Mutant viruses lacking a UL2 gene product appear to be compromised in their ability to reactivate from latency [42]. Given the quiescent state of the peripheral sensory ganglia, it is, therefore, surmised that UL2 is required for the maintenance of viral episomes in these cells [24,42]. UL2 may also play roles in the lytic phase of HHV-1 [42,79], which is substantiated by analysis of recent interactome data (including only virus-virus protein interactions; the data also includes indications for Ung interactions from other herpesvirus Subfamilies) [80]. There is no data on whether human UNG2 (the nuclear isoform of host cellular Ung) might also contribute to fitness of alphaherpesviruses. New approaches promise to shed light on replicative dynamics [81], thus questions such as these could well be answered in due course.

In betaherpesviruses, studies indicated that in HHV-5, Ung (the gene product of UL114) was essential for effective lytic phase initiation and also accelerated the rate of replication. It was also reported that UL114 is also a factor in the maintenance of HHV-5 latency [45,46,47]; a role for UL114 in lytic replication is also reported [48]. Not much is known about any role of Ung in *Roseoloviruses* (HHV-6A, HHV-6B, and HHV-7), but these viruses all encode an Ung with adaptive similarities to UL114 of HHV-5.

**Figure 6:**
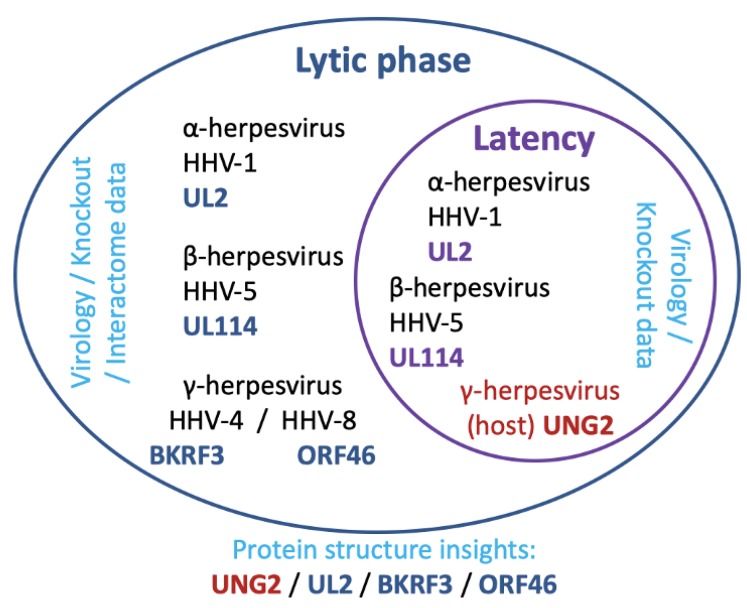
The latent and lytic phase of herpesviruses and relevance to uracil-DNA glycosylase. The simplified schematic depicts a cell cytosolic compartment (outer ellipse) with a nucleus (interior ellipse). Subfamily and virus are indicated in black text with uracil-DNA glycosylase encoded by that virus in blue text. Placement of text indicates the location UDG is currently thought to act (latent phase in the nucleus, and lytic phase in the cytosol) and the type of evidence supporting the premise (cyan text). Host cell encoded UNG2 is indicated with the named virus subfamily in dark red text, and placement indicates the phase of action (latency reactivation).

In gammaherpesviruses, the virus-encoded Ung (known as BKRF3 in HHV-4, and ORF46 in HHV-8) is likely to be an essential component of the lytic replisome in HHV-4 [49,50], and similarities in structural data supporting a possible mode for its interactions indicate this is probably the case also in HHV-8 (Figure 5) [51,52], and interestingly the human UNG2 isoform is known to be recruited to the maintenance of latency by the protein LANA in HHV-8 (Figure 6) [69].

## 12. A consideration of Specific Selectivity of Novel Chemical Entities for Herpesvirus Ung

Herpesvirus Ung, specifically the UL2 gene product of HHV-1, has previously been a subject of medicinal chemistry investigations [82,83,84,85,86]. Compounds were designed to take advantage of the exquisite specificity of Ung for a uracil base, and to develop series towards selectivity versus the host cell protein UNG2. The active site pocket of Ung is known, from nearly 200 structures deposited in the protein data bank to date, to be particularly well conserved in any example thus far studied from bacteria, to viruses and parasites, to eukaryotes. Nevertheless, preliminary medicinal chemistry research was able to develop compounds with an encouraging selectivity, albeit at sub-millimolar/ micromolar affinity [82,83]. More recently, it was described that a hydrophobic crevice runs from the active site in Ung, towards the protein core, explaining previous data; and, this could be taken advantage of to further refine specificity [86].

Considering the approach described, two obvious flaws present themselves when looking ahead to lead compound development. The first is that the starting point of these compounds is a nucleoside analogue. Therefore, given this is the basis of the current most successful antiherpetic drugs, it is unlikely that the need to obtain compounds with lower toxicity profiles than currently approved drugs would be fulfilled via such series. Second, the manner in which the nucleoside analogue is engineered for specificity into a hydrophobic cleft requires various lengths of aliphatic tails to be attached. General practice in drug design would probably choose to avoid the development of series via compounds likely to have very poor solubility characteristics from the outset. Therefore, this approach to the development of novel chemical entities for selective inhibition of herpesvirus Ung, versus the host enzyme, is unlikely to result in useful lead candidates.

The knowledge of specific differences between Ung of, in particular the β- and γ-herpesviruses, versus the canonical UNG2 of the host have matured since the aforementioned approach to Ung medicinal chemistry was examined. Structural biology has shown that in the Ung of γ-herpesviruses HHV-4 and HHV-8, there is a conserved novel structured elaboration of a critical DNA-binding loop essential for Ung substrate catalysis (Figure 5) [51,52]. Without this adaptation to the loop, it would appear that viral lytic phase replication is critically or fatally impaired in HHV-4 [50], and encouragingly independent data would suggest Ung is similarly indispensable also in the β-herpesvirus HHV-5 [45,46]. Whether the replicative roles of viral Ung will involve the differently adapted loop in the betaherpesvirus Subfamily, remains to be seen.

The loop elaboration in γ-herpesviruses is observed to be natively unstructured and to take up a rigidified form conserved in three dimensions when Ung is engaged with a DNA substrate [52]. The complex with duplex DNA is arranged such that it appears a signal is evolved in the DNA structure, relevant to replisome assembly [52]. This may be the reason why abrogation of the loop adaptation in herpersvirus Ungs ablates replication so effectively [50]. It is proposed that the near-surface location of this site, and the general mixed polar nature of the environs and within the loop structure itself, lends itself well to a structure-based drug design campaign (Figure 5) [41]. In fact protein engineering studies involving the Ung inhibitor protein SaUgi, which targets the Ung DNA binding cleft and minor groove DNA binding loop region show that impressive selectivity between Ung encoded by human, bacterial, α-herpesvirus and γ-herpesvirus can be demonstrated [76].

## 13. Summary and Conclusions

It is discussed that herpesviruses have retained and adapted a genomic copy of an Ung gene. The gene is apparently critical to viral fitness, and murine models lend further support that its absence leads to a rapid loss of viral fitness [87]. It is also noted that the host-encoded UNG2 may also be active, or an accessory to reactivation, in latency in the γ-herpesvirus HHV-8 [69]. Although considering the targeting of the host enzyme UNG2 may appear risky, a limited regimen that contributes to the weakening of the virus pool in an at-risk patient may be worth having access to. Inhibitors specific for UNG2 have been developed [88,89], which it is true are based on nucleotide chemistry; nevertheless targeting of the catalytic loop as discussed earlier [41,76], could give better scope for drug-like properties (i.e., lower hydrophobicity versus initial designs against Ung, and improvement of toxicity profiles versus current antiherpetics) likely to progress through lead optimisation to trials.

On another matter, functional conservation of exaptation within a subfamily (namely, the γ-herpesviruses) may be relevant to Ung function data within other subfamilies (namely, the β-herpesviruses) but structure-based drug design cannot be considered in the latter subfamily at the present time because no betaherpesvirus Ung structures have been deposited to date. It has been seen that good practice in drug discovery may still lead to late-stage drug candidate failures [8], but there is a need for compounds with good solubility characteristics, and lower toxicity, particularly in immune-compromised healthcare settings [1,2,4,5,6,7]. It is proposed that the Ungs of herpesviruses provide suitable ground to develop programmes that could reasonably deliver the required outcomes for future therapeutics.

## Figures and Tables

**Table 1:** Pathogenic herpesviruses of humans *.

Virus Common Name	Virus Numeric Name	*Herpesviridae* Subfamily	Reference Genome Accession Code	Genome Length	Uracil-DNA Glycosylase Accession Code
HSV-1	HHV-1	*Alphaherpesvirinae*	NC_001806	152222	YP_009137076
HSV-2	HHV-2		NC_001798	154675	YP_009137153
VZV	HHV-3		NC_001348	124884	NP_040181
HCMV	HHV-5	*Betaherpesvirinae*	NC_006273	235646	YP_081554
-	HHV-6A		NC_001664	159378	NP_042974
-	HHV-6B		NC_000898	162114	NP_050260
-	HHV-7		NC_001716	153080	YP_073819
EBV	HHV-4 **	*Gammaherpesvirinae*	NC_007605	171823	YP_401679
KSHV	HHV-8		NC_009333	137969	YP_001129398

* The table represents major human pathogens with complete genome records, but does not include zoonotic viruses. ** HHV-4 Type 2 is not included in this table. The dash symbol in the leftmost column indicates there is no common or colloquial name, other than descriptive, e.g. Roseola [causing] virus.

**Table 2:** Perturbation of DNA fidelity and cellular regulation by pyrimidine deamination events.

Canonical State	Protective Factors	Post-Error State	Consequence of Error	Repair Measure
C:G base-pair	dsDNA state or protein complex	U:G mistmatch	promutagenic	UDG
^5-me^ C:G base-pair	dsDNA state or protein complex	T:G mismatch	promutagenic	TDG
T:A base-pair	dUTPase and DNA polymerase	U:A base-pair	dysregulatory	UDG
